# Animal and Vegetable Pathology

**Published:** 1901-09-07

**Authors:** 


### ANIMAL AND VEGETABLE PATHOLOGY.

In a clinical lecture recently delivered at the London Hospital Mr. Jonathan Hutchinson[1] pointed out some of the lessons to be learnt from vegetable pathology. Referring to the importance of wounds as giving ingress to infection, he said that a wound to the bark of a plant would allow a fungus to settle on it and grow in it and set up such disease of the stem as to cause the plant to rot. The original injury might be quite a small affair produced by a bird or a hailstone or a blow, but that slight injury would allow the fungus to enter, and that fungus having passed into the wound would pass into the stem of the tree and would probably grow and work its way up its long axis. Another kind of fungus which destroys fir trees on a large scale enters by the roots and is believed to be spread by the agency of rats and rabbits, which burrowing and eating the roots injure them—only a little, perhaps, but by passing from tree to tree and carrying the fungus with them, they infect the root and kill the tree. Thus we see examples of disease in plants being spread by infection just as in animals. After instancing many other examples of the similarities and differences between animal and vegetable pathology, Mr. Hutchinson went on to give a warning that we must not assert too positively, that diseases which we find to be caused by apparently different organisms must of necessity be independent and specific. A great lesson in caution on this point, he said, was to be derived from the old doctrine as regards rust on wheat. Rust is a disease in which the leaf of the wheat becomes brown all over and dies prematurely, and it became an urgent question with agriculturalists as to how it could be prevented. Farmers observed that where the common barberry grew in hedges there was likely to be rust in the wheat. Botanists were asked whether there was any causal connection between these two, and they at once declared that such a thing was impossible. They said they recognised the fungus which grew on the barberry, and that they also recognised that which was produced on the wheat, and they maintained that it was impossible that they could be the same, as they belonged to totally different species. The farmers, however, still protested that where barberry grew there was the rust, and notwithstanding the opinion of the botanists, they felt that there was some connection between the two, and they acted upon their belief by getting rid of the barberry trees. A law was passed in the State of Massachusetts, calling upon farmers to exterminate all barberry trees out of their hedges. This was made compulsory, and if the farmers did not do it the work was to be done by local authorities, and they were to be charged with the cost. All this time the scientific men were saying that the farmers were ignoramuses who did not study botany, and did not understand the matter, because the fungi on the two plants were totally distinct. And then came the great discovery that this fungus is one which requires two different hosts; in one host it produces one special spore and in another another spore. It is the same fungus, which developing on the barberry and then being blown off takes root on the wheat and becomes spread over the wheatfields as rust. The farmers were shown to be right, and these apparently different fungi were really the same in different stages of its development. Thus science lagged behind the observations of practical men. We must be careful then not to urge too strongly theories which seem contradicted by the general verdict of experience. Perhaps we may have to simplify our conceptions, for we may find that many of the diseases which we have considered as separate are not in reality so.

[1] Lancet, August 31.

